# On Coryza Maligna

**Published:** 1860-06

**Authors:** Bernard Kelly

**Affiliations:** Physician to the New York Dispensary


*On Coryza Maligna.* By BERNARD KELLY, M.D., Physician to the New York Dispensary.

J. C., at seven months, was attacked on the 13th of April last with coryza maligna. I saw the child on the following day, and consequently had ample opportunity to observe the peculiar phenomena of the disease almost from its origin to its fatal termination. The symptoms simulated those of ordinary nasal catarrh, but were marked by a greater intensity. There was an abundant discharge of a glairy mucus from the nostrils; the conjunctivæ were injected; the face and neck sensibly swollen; the temperature of the skin was very little, if any, above the natural standard; a gentle perspiration covered the whole body; the bowels showed a slight tendency to constipation. The little patient nursed with a great deal of difficulty and distress, being obliged to relinquish the nipple every two or three seconds in order to catch breath. He was by no means fretful, as one might, at first sight, expect from the excessive gravity of the case, but, on the contrary, lay, for the most part of the time, in a torpid, drowsy condition, from which he was only aroused by the assiduous attentions of his nurse, or when excited by the cravings of hunger. There was a constant exacerbation of all the symptoms towards night.

Severe, unquestionably, as the latter were, I did not venture to have recourse to decided antiphlogistic measures. I calculated on the disease persisting a considerable time, and consequently deemed it more judicious to foster the strength of the little sufferer, in order to conduct him safely, if possible, through the terrible ordeal which awaited him. I ordered a quarter of a grain of quinine to be given every three hours, with a powder composed of *hydrarg. cum creta*, and rhubarb, at bedtime, in sufficient proportions to relax the bowels gently. This treatment was persevered in for three days with every hope of encouragement, when the symptoms suddenly redoubled in severity. It was now deemed advisable to call a consultation, and accordingly a physician of great experience and judgment was sent for, who concurred in the treatment, and suggested the propriety of a warm bath that night. It was given, and the following morning the child was covered with a profuse eruption of measles. It may be well to remark here, that there was no cough, nor any physical sign of bronchitis or pulmonary congestion previous to, or following the appearance of the exanthem. The breathing was natural in every respect, save that it was performed entirely through the mouth, which was thereby parched, and needed frequent wetting. The catarrhal symptoms abated not a particle in their intensity; the general condition of the patient, however, was not materially worse. His food consisted principally of arrow-root and beef-tea, which he used with no inconsiderable relish. Towards the eighth day from the first attack, the eruption began to decline, and the discharge from the nares to grow less consistent, but scarcely less profuse. He again sought the breast with great avidity (for several days had now elapsed since he was capable of nursing,) and bore the spoon-feeding with evident reluctance. His attempts, however, were not more successful than those mentioned in the early stage of the disease, and precisely for the same reason. The bowels now became excessively relaxed, without any apparent cause, and the strength of the child was sinking rapidly. Small doses of *Tr. opii camph.* were given with the quinine, but were obliged to be suspended from the fact of nausea and vomiting being induced. The alvine discharges became less frequent and watery; they were, however, exceedingly fœtid—a character which even the breath and cutaneous exhalation partook of to a certain degree.

Matters continued in this precarious state till about the fourteenth day, when all the symptoms of membranous croup suddenly manifested themselves. The respiration became hurried and stridulous; while ever and anon the *brazen cough* rang upon the ear with an ill-boding sound. No febrile excitement supervened; on the contrary, the temperature of the surface (which was now of a sallow, dingy hue) was very sensibly diminished. All the animal heat of the patient seemed to concentrate itself in the soles of the feet and palms of the hands. On examining the fauces attentively, the whole soft palate and tonsils were found covered with a yellowish-white false membrane; the nares were lined by a similar deposit. The disease had marched so rapidly (as shown by urgent *dyspnœa* and coldness of the body) that I utterly despaired of all topical medication. The chlorate of potash was now given with the view of arresting the further exudation of lymph and oxydizing the blood; while the patient's strength was sought to be supported, in the mean time, by aromatic spirits of ammonia and burnt brandy. All these means proved completely inadequate; for he sank steadily, and died in twenty-four hours from the first manifestation of the croupal symptoms. There were no convulsions from the beginning to the close of the disease. The child preserved throughout his natural intelligence, which, I may remark, was unusually precocious.

Were I to express an opinion candidly upon the pathology of *coryza maligna*, I would unhesitatingly place it in the same category with scarlatina, erysipelas, diphtherite, and *à fortiori* with croup. The same depression of the vital powers, the same blood-poisoning, I might add, seem to be at the source of all, and to constitute their most prominent morbid characters. I should, therefore, were I to meet another case similar to the one under consideration, begin from the very outset (so as to crush it in the bud) with free doses of quinine and tincture of iron, either in combination and simultaneously, or separately and in alternating intervals, as its peculiarity or emergency might require. The tender age of the patient should present no obstacle to a bold trial of this treatment. For there is very little difference (as far as the life of the subject is concerned) whether we calmly look on with folded arms, as it were, and allow the disease to march to a fatal termination, or that we arrive at the same end through the activity of our remedies; such, at least, are my present convictions; further observations may lead to more important developments.

The most reliable topical applications would, undoubtedly, be the sulphate of alum and nitrate of silver; yet, if the disease consists in some fundamental change (as I believe it does) in the physiological constitution of the blood, it is not very easy to comprehend how local means can be of any decided benefit, while the *materies morbi* lies so far beyond their reach, and thereby remains intact. The physician, now-a-days, would be rationally deemed behind his age, who would rely upon topical applications solely in the treatment of erysipelas or scarlatina; and, reasoning from analogy, there cannot be the slightest doubt but that the same rule would apply equally appropriate in the case of coryza maligna. All that may well be expected from injections of alum or nitrate of silver is an arrest of further exudation of lymph, or its destruction, if already effused, in the parts with which they come in contact, and thus become auxiliary to the constitutional treatment; but the mucous membrane of the larynx, trachea, and bronchial tubes yet remains, to furnish a large surface for the fatal scope of the disease, and will, consequently, circumscribe the salutary action of local applications within very narrow limits.

